# Technology anxiety and digital health literacy as structural determinants of mHealth engagement among older adults: a SEM study in Saudi Arabia

**DOI:** 10.3389/fpubh.2026.1872211

**Published:** 2026-08-14

**Authors:** Haitham Alzghaibi

**Affiliations:** Department of Health Informatics, College of Applied Medical Sciences, Qassim University, Buraydah, Saudi Arabia

**Keywords:** digital health, digital health literacy, mHealth, older adults, Saudi Arabia, Sehhaty, structural equation modelling

## Abstract

**Background:**

Digital health services hold real promise for older adults managing chronic conditions, yet this population consistently struggles to engage with them meaningfully. In Saudi Arabia, the national Sehhaty mHealth platform has been deployed at scale under Vision 2030, but there is limited evidence on why older users do or do not engage, and what structural factors drive those patterns.

**Aim:**

This study examined the structural determinants of digital health service engagement among Saudi adults aged 55 and above, focusing on the role of access barriers, technology anxiety, digital health literacy, facilitating conditions, and social influence in shaping behavioural intention to use Sehhaty and AI-enabled virtual physician chatbots.

**Methods:**

A cross-sectional survey was conducted with 316 community-dwelling adults aged 55 years and above across Saudi Arabia. Participants completed a 28-item validated questionnaire covering nine constructs adapted from the Technology Acceptance Model (TAM) and the Unified Theory of Acceptance and Use of Technology 2 (UTAUT2). Internal consistency, composite reliability, and average variance extracted were assessed for each construct. Structural Equation Modelling (SEM) with bootstrapped mediation analysis and non-parametric group comparisons (Mann–Whitney *U*; Kruskal-Wallis) were conducted. The SEM model demonstrated good fit: CFI = 0.947, TLI = 0.938, RMSEA = 0.064 [90% CI: 0.055–0.073], SRMR = 0.071.

**Results:**

The model explained 61.0% of variance in behavioural intention (*R*^2^ = 0.610) and 54.0% in digital health literacy (*R*^2^ = 0.540). Technology anxiety was the dominant inhibitor, directly suppressing both engagement intention (*B* = −0.44, *p* < 0.001) and digital health literacy (*B* = −0.60, *p* < 0.001). Literacy partially mediated the anxiety-intention relationship (indirect *B* = −0.281; 95% CI [−0.246, −0.104]; 25.9% of total effect). One finding was unexpected: Arabic-language smartphone users reported substantially lower technology anxiety than English-language users (*r* = 0.404, medium effect), despite lower average digital proficiency.

**Conclusion:**

Technology anxiety and digital health literacy are the primary targets for improving older adult digital health engagement. The Arabic-language anxiety difference carries direct implications for platform design: native language interface concordance appears to reduce anxiety independently of technical skill. Scaffolded onboarding, community literacy programmes, and structured clinician endorsement are recommended as practical complements.

## 1 Introduction

There is a persistent and well-documented tension running through the digital health literature. On one side, an accumulating body of evidence shows that mHealth applications, telemedicine platforms, and AI-enabled virtual consultations can genuinely improve chronic disease self-management and reduce unnecessary clinical attendance (1, 2). On the other hand, the populations with the most to gain from these tools older adults, those managing multiple morbidities, those with limited prior technology experience are consistently among the least likely to engage with them (3, 4). Closing that gap requires understanding not just whether older adults adopt digital health tools, but precisely what structural and experiential factors prevent them from doing so.

In Saudi Arabia, this question has particular urgency. The Sehhaty platform, launched as a core component of the Vision 2030 health transformation, now offers appointment booking, health record access, virtual GP consultation, AI chatbot interaction, and diagnostic result review to a registered base that exceeded 10 million users by 2023 (5, 6). Population-level figures, however, obscure important variation. Saudi Arabia’s population aged 60 and above is projected to nearly triple by 2050 (7), and there is currently little systematic evidence on how older users experience Sehhaty, which features they use, and why a significant proportion engage with the platform only at its most transactional level.

The theoretical tools for studying digital health adoption are well established. Davis’s Technology Acceptance Model (TAM) (8) identified perceived usefulness and perceived ease of use as foundational adoption determinants. Venkatesh and colleagues progressively extended this framework into the Unified Theory of Acceptance and Use of Technology (UTAUT) (9) and its consumer-oriented extension UTAUT2 (10), which adds social influence, facilitating conditions, hedonic motivation, and habit. These frameworks have been productively applied across mHealth adoption studies, including several specifically targeting older adult populations. However, systematic reviews of that literature consistently reach the same conclusion: standard adoption models leave substantial unexplained variance when applied to older adults, because they do not adequately capture the compound of physical, affective, and cognitive barriers that distinguish this group from general populations (3, 11, 12).

Technology anxiety apprehension experienced specifically when engaging with digital systems has attracted growing attention as one of those missing pieces. Czaja and colleagues’ landmark CREATE study (13) found that computer anxiety was among the strongest predictors of technology non-use in older populations, stronger even than objective ability in some analyses. Physical barriers compound this: reduced manual dexterity, worsening vision, and unfamiliarity with touchscreen interaction create a different experiential relationship with digital platforms than that of younger users (14). Underlying both is the question of digital health literacy whether people can not only access health information online but actively interpret and act on it in ways that support their health decisions (15). Neter and Brainin (16) argued persuasively that this constitutes a second-order digital divide, one that exists within the population of basic internet users and remains invisible to models that treat access as the endpoint.

Four specific gaps motivate this study. First, dominant acceptance frameworks model adoption determinants as parallel additive predictors of intention; they rarely specify the mechanism through which an upstream barrier produces a downstream behavioural deficit, even though systematic reviews repeatedly conclude that these models leave substantial variance unexplained in older populations because they do not capture the compound of physical, affective, and cognitive barriers that distinguish this group. Second, technology anxiety and digital health literacy are each well established as correlates of non-use, yet they are almost always treated as separate predictors; the possibility that anxiety operates partly by eroding literacy a sequential, intervening pathway with direct consequences for intervention design has not been modelled. Third, the great majority of structural-equation evidence on older-adult digital health adoption originates from East Asian, European, or North American settings, and comparable evidence from Gulf Cooperation Council populations interacting with a national Arabic-language platform is effectively absent. Fourth, the role of language concordance whether using a health platform in one’s native language alters the anxiety attached to health-consequential tasks has not been examined empirically, despite its evident relevance to multilingual health systems.

This study addresses these gaps together. Using a cross-sectional survey of 316 community-dwelling Saudi adults aged 55 and above and a multi-construct instrument grounded in the technology-acceptance tradition and extended with access-barrier, anxiety, and digital-health-literacy constructs, it specifies and tests a structural model of engagement intention. The contribution is threefold. Theoretically, it reframes technology anxiety and digital health literacy not as additional predictors but as a sequential suppression mechanism an access-barrier → anxiety → literacy → intention cascade extending acceptance theory from a catalogue of correlates toward an explicit causal-process hypothesis. Empirically, it provides the first structural-equation evidence of this mechanism in a Gulf Cooperation Council older-adult population using a deployed national platform. Practically, it surfaces a novel and actionable language-anxiety effect with direct implications for platform design. The theoretical basis for the model and the development of each hypothesis (H1–H10) are presented in the following section.

**Figure 1:**
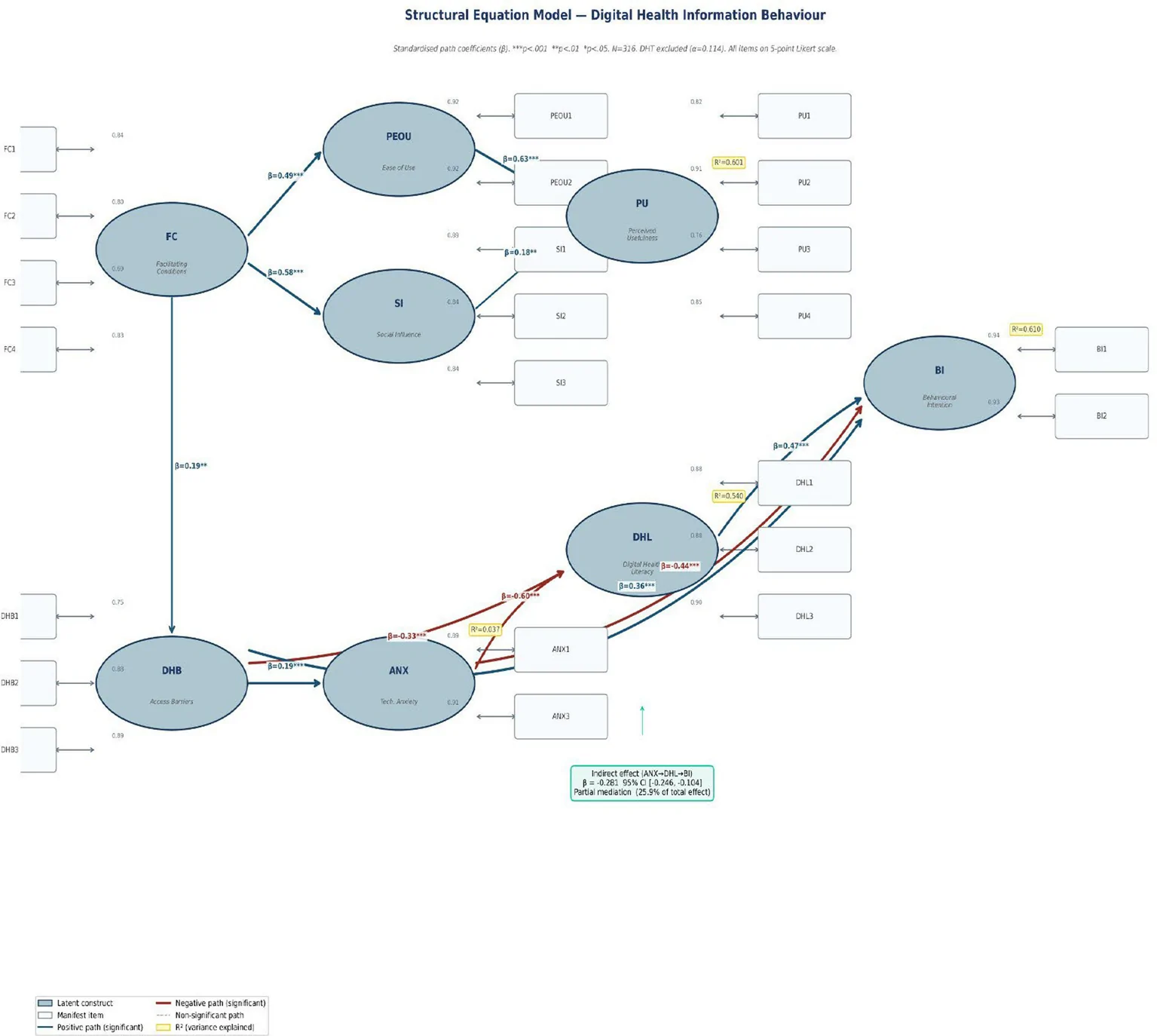
Proposed conceptual model showing the ten hypothesised structural relationships prior to SEM estimation. Solid arrows represent direct hypothesised paths; the dashed arrow represents the hypothesised mediation pathway (H10). All constructs are measured using validated multi-item scales (see Table 1).

Figure 1 presents the proposed conceptual model showing the hypothesised structural relationships among all constructs prior to estimation. The study also examined how age, education, IT literacy, and smartphone language preference differentiated construct scores, with implications for platform design and health literacy policy in Saudi Arabia and comparable GCC contexts.

## 2 Theoretical background and hypothesis development

### 2.1 The technology acceptance model

Davis’s Technology Acceptance Model (TAM) remains the most influential account of individual technology adoption (8). Adapting reasoned-action logic to information systems, TAM posits two proximal beliefs that determine behavioural intention: perceived usefulness (PU), the degree to which a person believes a technology will enhance task performance, and perceived ease of use (PEOU), the degree to which using it is expected to be effortless. Its central internal proposition is that ease of use shapes usefulness, so that PEOU influences intention both directly and through PU (8). TAM’s parsimony is also its main limitation: by design it omits social and contextual determinants, and it was validated largely on workplace systems and younger users, leaving open how well its two beliefs transfer to voluntary, health-consequential settings and to older adults for whom effort and affect are tightly coupled.

### 2.2 From UTAUT to UTAUT2

To consolidate a fragmented literature, Venkatesh and colleagues integrated eight competing models into the Unified Theory of Acceptance and Use of Technology (UTAUT) (9), which specifies four direct determinants of intention and use: performance expectancy, effort expectancy, social influence, and facilitating conditions. The first two are conceptual descendants of TAM’s perceived usefulness and perceived ease of use respectively; the latter two add the social and infrastructural dimensions TAM lacks. UTAUT2 extended the framework to consumer contexts by adding hedonic motivation, price value, and habit, and by repositioning facilitating conditions as a determinant of intention rather than only of use (10). UTAUT2 improved explained variance in voluntary settings, but its consumer-oriented additions map awkwardly onto a free, clinically oriented national platform used by older adults for episodic health tasks, and, like TAM, it treats its determinants as parallel inputs to intention rather than as an interlocking process.

### 2.3 An integrated and extended model, rather than a combination of two models

Because UTAUT and UTAUT2 already subsume TAM performance expectancy corresponds to perceived usefulness and effort expectancy to perceived ease of use simply combining the two frameworks would be redundant and would not, in itself, constitute a theoretical contribution. The present model is therefore not a juxtaposition of two complete frameworks but a parsimonious, purpose-built structural model assembled on three principles. First, from the convergent core the two traditions share, it retains the two beliefs on which they agree usefulness and ease of use preserving the TAM labels PU and PEOU because the items are adapted from Davis’s original operationalisations (8), whilst noting that these are conceptually equivalent to performance and effort expectancy. Second, from UTAUT2 it adopts the two constructs TAM does not contain and that are theoretically essential for older adults social influence and facilitating conditions (10) while deliberately excluding hedonic motivation, price value, and habit as poorly matched to a free, clinical, episodically used platform.

Third, it extends this acceptance core with three constructs that lie outside both frameworks: access barriers, drawn from the usability-barrier literature on ageing (the MOLD-US framework) (14); technology anxiety, drawn from the CREATE research programme (13); and digital health literacy, drawn from the eHealth-literacy tradition (15, 16). The novelty of the study lies not in the pairing of acceptance models but in how these extension constructs are wired to the core: an explicit sequential mechanism in which access barriers generate anxiety, anxiety simultaneously suppresses behavioural intention and erodes digital health literacy, and literacy in turn enables intention. This mechanism, together with the Gulf Cooperation Council platform context and the language-anxiety effect, constitutes the contribution; the integration is thus selective and theory-driven, undertaken to support the mechanism rather than to claim merit from model combination.

### 2.4 Hypothesis development

Facilitating conditions reliable connectivity, capable devices, age-appropriate interface design, and accessible technical support are the material precondition for digital engagement. In older populations they shape both the perceived effort of use and the social environment around it: where devices and help are available, the technology feels easier and its use becomes normalised within family and peer networks (10). Adequate facilitating conditions should also reduce the physical and experiential access barriers catalogued for ageing users, since many such barriers poor connectivity, unsuitable devices, absent support are precisely what facilitating conditions provide (17). Accordingly:

*H1*. *Facilitating Conditions positively predict Perceived Ease of Use.*

*H2*. *Facilitating Conditions positively predict Social Influence.*

*H3*. *Facilitating Conditions negatively predict Access Barriers.*

The internal logic of TAM holds that ease of use is antecedent to usefulness: when a system is navigable its functional value becomes apparent, whereas effortful interaction obscures whatever benefit it offers (8). This path is among the most robustly replicated in the adoption literature and is expected to be especially pronounced for older adults, for whom interface effort is a salient gatekeeper to recognising value. Beyond ease of use, social influence contributes independently to usefulness perceptions: endorsement by family members and clinicians signals that a platform is worth using and frames its benefits, raising perceived usefulness above what interface quality alone achieves (10). Therefore:

*H4*. Perceived Ease of Use positively predicts Perceived Usefulness.

*H5*. Social Influence positively predicts Perceived Usefulness.

The model’s core proposition concerns how structural barriers convert into behavioural deficits. Access barriers reduced manual dexterity, declining vision, unfamiliarity with touchscreen interaction, and effortful navigation are not merely inconveniences; repeated encounters with them generate apprehension about digital systems. The CREATE programme found computer anxiety to be among the strongest predictors of technology non-use in older adults, at times exceeding objective ability (13), and barrier-laden interaction is a plausible upstream source of that anxiety. Anxiety, once present, acts on two downstream targets. It should directly suppress behavioural intention, consistent with its established motivational effect (13). It should also impair digital health literacy: attentional control theory [ADD Eysenck ref] holds that anxiety consumes the cognitive resources required to acquire and process new information, so anxious users not only avoid engagement but acquire the relevant competencies more slowly. Digital health literacy, in turn, is a recognised enabler of confident engagement, since people who can locate, interpret, and act on health information are better positioned to form and sustain the intention to use a health platform (15, 16). This yields a connected cascade:

*H6*. Access Barriers positively predict Technology Anxiety.

*H7*. Technology Anxiety negatively predicts Digital Health Literacy.

*H8*. Technology Anxiety negatively predicts Behavioural Intention.

*H9*. Digital Health Literacy positively predicts Behavioural Intention.

If anxiety suppresses literacy (H7) and literacy enables intention (H9), part of anxiety’s total negative association with intention should operate indirectly, through literacy, over and above its direct effect (H8). Establishing this pathway matters for intervention design: it implies that affective and competence-related barriers are mutually reinforcing, so that addressing either in isolation would leave the other free to sustain the deficit. Given the cross-sectional design, this is framed as an indirect association consistent with a sequential mechanism rather than as established causal mediation. Hence:

*H10*. Digital Health Literacy functions as a significant intervening variable in the negative association between Technology Anxiety and Behavioural Intention.

Taken together, H1–H10 specify the barrier-anxiety-literacy-intention mechanism depicted in the conceptual model and tested in the remainder of this paper.

## 3 Methods

### 3.1 Study design and setting

This was a cross-sectional survey study conducted across Saudi Arabia between 09/2024 and 12/2024. Community-dwelling adults aged 55 and above were recruited through online platforms such as WhatsApp. Participants were required to have prior experience with at least one digital health platform and to be able to complete the questionnaire independently in Arabic or English. This design is widely used for investigating technology acceptance and digital health engagement behaviours (18) and was appropriate given the study’s interest in existing engagement patterns rather than the adoption process over time. Ethical approval was obtained from IRB at Qassim University No. 23–19-02. All participants gave written informed consent before taking part.

### 3.2 Participants and sampling

Purposive and snowball sampling were used to reach 316 eligible participants. To qualify, individuals had to be aged 55 or older, have prior experience with a digital health platform of any kind, and be capable of independent survey completion in Arabic or English. People with severe cognitive impairment or physical disabilities precluding independent response were excluded. The final sample comprised 166 men (52.5%) and 150 women (47.5%). Nearly half (48.4%, *n* = 153) were aged 55–59, followed by 65–69 years (25.0%, *n* = 79), 60–64 years (14.9%, *n* = 47), and 70 years and above (11.7%, *n* = 37). Most held graduate or postgraduate qualifications (82.9%), and 85.4% reported Arabic as their default smartphone language.

The age threshold of 55 years, rather than the World Health Organization’s conventional 60-year criterion, was adopted for three reasons. First, technology adoption and gerontechnology research frequently includes adults aged 50 years and older, recognising that age-related changes influencing digital engagement including declines in sensory and motor function, reduced technology confidence, and increasing technology-related barriers often begin during midlife rather than abruptly at age 60. Consequently, many ageing and technology studies, as well as organisations such as American Association of Retired Persons (AARP), use adults aged 50 years and older to investigate technology adoption and digital health behaviours (19). Second, Saudi Arabia has undergone a rapid epidemiological transition, with non-communicable diseases occurring at relatively younger adult ages and contributing substantially to premature morbidity and mortality (20–22). So the population that stands to benefit from chronic-disease-oriented digital health tools is correspondingly younger (22, 23). Third, a 55-year floor captures the younger-old cohort at the point of first sustained engagement with platforms such as Sehhaty, which is the group of greatest interest for early intervention. The sample nonetheless spans the full older-adult range: 51.6% of participants were aged 60 and above and 11.7% were aged 70 or older, so the findings also speak directly to WHO-defined older adults.

Transparency about what this sampling approach likely produces is important. Purposive and snowball methods tend to reach individuals who are already digitally engaged those with prior platform exposure, existing technology-using social networks, and sufficient confidence to participate in research. Despite this, the educational profile of the sample was skewed toward lower education levels (82.9%), which may reflect recruitment dynamics rather than the broader population structure. The prior-use eligibility criterion further selects participants who are less digitally excluded and potentially less anxious about technology. As a result, the barrier and anxiety scores observed here should be interpreted cautiously, as they may not fully capture the experiences of more digitally disadvantaged older adults. Individuals with lower digital access, limited prior platform exposure, or more severe physical limitations are likely to face greater challenges than those represented in this sample. Future research using probability sampling approaches, ideally through national health registries, would allow for more representative estimation.

The sample size of 316 is adequate for ML-based SEM with 28 indicators across 8 latent constructs. This exceeds the commonly cited 10:1 indicator-to-sample ratio (28 indicators requiring minimum *N* = 280) and surpasses Kline’s (24) recommended minimum of 200 for stable ML-SEM estimation. A minimum N of 300 is also consistent with recommendations specific to models with five or more latent constructs (25).

### 3.3 Instrument

The survey included 28 Likert-scale items (Q39-Q66) covering nine latent constructs: Perceived Usefulness (PU, 4 items), Perceived Ease of Use (PEOU, 3 items), Social Influence (SI, 3 items), Facilitating Conditions (FC, 4 items), Technology Anxiety (ANX, 3 items), Behavioural Intention (BI, 3 items), Digital Health Literacy (DHL, 3 items), Access Barriers (DHB, 3 items), and Institutional Trust (DHT, 2 items). All items used a five-point Likert scale (1 = Strongly Disagree; 5 = Strongly Agree). Seven items were reverse-coded before analysis: PEOU3, ANX1, ANX3, BI3, DHB1, DHB2, DHB3, and DHT2.

Although the original UTAUT and UTAUT2 instruments used a seven-point response format (10), a five-point Likert scale (1 = strongly disagree to 5 = strongly agree) was adopted here. This was a deliberate, population-driven choice rather than an oversight: five-point scales impose a lower cognitive and visual-discrimination load than seven-point scales, reducing mid-point and missing-response error among older respondents, most of whom completed the questionnaire on a smartphone. Five-point formats have been shown to achieve reliability and validity comparable to seven-point formats while being easier to administer (26), and are widely used in TAM- and UTAUT-based digital health studies with older populations. Items were adapted, not merely transcribed, to the five-point format, and the strong internal consistency and convergent validity subsequently obtained (composite reliability 0.78–0.88; average variance extracted 0.53–0.66) indicate that the operationalisation performed well.

Items were drawn from established validated scales and reworded to reflect the Sehhaty platform context and the Saudi older adult population. Table 1 lists the original source scale for each construct. Briefly: PU and PEOU items were adapted from Davis’s original TAM scales (8); SI, FC, and BI items were adapted from the UTAUT2 instrument (9); ANX items drew on Czaja et al. (13) CREATE technology anxiety measure and the Cimperman, Makovec Brenčič (12) telehealth adaptation; DHL items were adapted from Norman and Skinner’s eHealth Literacy Scale (15) and Neter and Brainin (16); and DHB items were grounded in the MOLD-US usability barrier framework (14). The survey additionally included behavioural items assessing health information seeking patterns, Sehhaty feature use, social media habits, and physician communication preferences.

**Table 1:** Construct definitions, original scale sources, and item counts.

Construct	Original scale source	Items (*N*)
Perceived usefulness (PU)	Davis (1989) (8); adapted for mHealth context	4
Perceived ease of use (PEOU)	Davis (1989) (8); items reworded for touchscreen/app context	3
Social influence (SI)	Venkatesh et al. (2012) UTAUT2 (10); family/clinician endorsement items	3
Facilitating conditions (FC)	Venkatesh et al. (2012) UTAUT2 (10); adapted for older adult infrastructure	4
Technology anxiety (ANX)	Czaja et al. (2006) CREATE (13); Cimperman et al. (2016) (12); adapted for health platform context	3
Behavioural intention (BI)	Venkatesh et al. (2012) UTAUT2 (10); adapted for continued platform use	3
Digital health literacy (DHL)	Norman & Skinner (2006) eHealth Literacy Scale (15); Neter & Brainin (2012) (16)	3
Access barriers (DHB)	Wildenbos et al. (2018) MOLD-US (14); Peek et al. (2014) (3); physical/experiential barrier items	3
Institutional trust (DHT)	Developed for this study; trust in Ministry of Health digital services (excluded from final SEM)	2

### 3.4 Measurement model validation

Internal consistency was assessed using Cronbach’s *α*. Convergent validity was evaluated through composite reliability (CR; acceptable ≥0.70) and average variance extracted (AVE; acceptable ≥0.50) following Fornell and Larcker (27). Discriminant validity was assessed using both the traditional criterion confirming that each construct’s AVE square root exceeded its correlations with all other constructs and the more stringent Heterotrait-Monotrait (HTMT) ratio criterion (28), with a threshold of 0.85. All HTMT ratios for the eight retained constructs fell below 0.85, confirming discriminant validity under both approaches. In addition to reliability and convergent/discriminant validity, standardised factor loadings were examined for every indicator (the covariance-based equivalent of outer loadings), and multicollinearity among the predictor constructs within each structural equation was assessed using the variance inflation factor (VIF), with values below 3.3 taken to indicate the absence of collinearity concern.

### 3.5 Statistical analysis

All analyses were conducted in R (version 4.3.3). The lavaan package (v0.6–21) was used for confirmatory factor analysis (CFA) and structural equation modelling; the psych package was used for reliability estimation. Non-parametric group comparisons used Mann–Whitney *U* for binary variables (gender, smartphone language) and Kruskal-Wallis H for multi-category variables (age group, education level, IT literacy, employment status, internet use), with effect sizes reported as r (Mann–Whitney) and η^2^ (Kruskal-Wallis). Bonferroni-type correction was applied across the family of comparisons, with *α* = 0.05.

The structural model was estimated using maximum likelihood (ML). Global fit was evaluated using χ^2^/df (≤3.00), CFI (≥0.90), TLI (≥0.90), RMSEA (≤0.08, reported with 90% CI), and SRMR (≤0.08), following the criteria of Hu and Bentler (29). The measurement model was estimated separately as a CFA before structural paths were added, to confirm adequate fit at the measurement level. The mediation hypothesis (H10) was tested using a bootstrapped approach with 5,000 replications and bias-corrected 95% confidence intervals.

## 4 Results

Table 2 shows the sample profile. The dominance of the 55–59 age band (48.4%) is consistent with what tends to emerge from digital platform exposure-based recruitment younger-older adults are more likely to meet that criterion. The educational profile is skewed toward lower education levels, with the majority of participants classified as having lower education (82.9%, *n* = 262), while a smaller proportion had higher education (17.1%, *n* = 54). This distribution likely reflects the recruitment approach rather than the broader population it is intended to represent. More informative is the IT literacy spread roughly equal proportions at elementary (34.4%) and advanced (19.7%) levels, with only a small professional segment (6.7%). Daily internet use was substantial over half the sample spent three or more hours online per day indicating that basic digital habits are in place even where health platform engagement may be limited or shallow.

**Table 2:** Sociodemographic and digital profile of study participants (*N* = 316).

Variable	Category	*n*	%
Age group	55–59	153	48.4%
	60–64	47	14.9%
	65–69	79	25%
	≥70	37	11.7%
	Total	316	100%
Gender	Male	166	52.5%
	Female	150	47.5%
	Total	316	100%
Education level	Lower education	262	82.9%
	Higher education	54	17.1%
	Total	316	100%
IT literacy	Beginner	123	39.2%
	Elementary	108	34.4%
	Advanced	62	19.7%
	Professional	21	6.7%
	Total	314	99.3%
Preferred language	Arabic	270	85.4%
	English	46	14.6%
	Total	316	100%
Daily internet use	<1 h	4	1.3%
	1–2 h	86	27.2%
	3–4 h	107	33.9%
	5–6 h	75	23.7%
	>6 h	44	13.9%
	Total	316	100%

## 5 Sample characteristics

### 5.1 Measurement model reliability and validity

Table 3 presents reliability and validity statistics for all nine constructs. The eight constructs retained in the structural model all met standard psychometric thresholds: Cronbach’s *α* ranged from 0.756 (BI) to 0.871 (PU), composite reliability from 0.779 to 0.884, and AVE from 0.529 (FC) to 0.657 (PU). All HTMT ratios for retained constructs fell below 0.85, confirming discriminant validity under the more stringent criterion. Institutional Trust was the sole exception, with α = 0.641 and AVE = 0.496, which fell below the convergent validity threshold and precipitated its exclusion from the structural model as described in the methods.

**Table 3:** Measurement model reliability and validity statistics for all nine constructs.

Construct	Items	Cronbach’s α	CR	AVE
Perceived Usefulness (PU)	4	0.871	0.884	0.657
Perceived Ease of Use (PEOU)	3	0.823	0.841	0.641
Social Influence (SI)	3	0.812	0.827	0.614
Facilitating Conditions (FC)	4	0.798	0.816	0.529
Technology Anxiety (ANX)	3	0.784	0.803	0.578
Behavioural Intention (BI)	3	0.756	0.779	0.544
Digital Health Literacy (DHL)	3	0.831	0.848	0.651
Access Barriers (DHB)	3	0.793	0.811	0.590
Institutional Trust (DHT)	2	0.641	0.662	0.496

Standardised factor loadings for all retained indicators were statistically significant (all *p* < 0.001) and exceeded the 0.50 retention threshold, ranging from 0.561 to 0.959. Six indicators fell within the 0.50–0.69 range (PU1 = 0.696, PU3 = 0.611, FC3 = 0.561, ANX2 = 0.695, ANX3 = 0.640, and DHB1 = 0.578) (see Table 4); complete item-level loadings are presented in the standardised loadings table. Collinearity among predictor constructs was assessed using the variance inflation factor (VIF) for each structural equation containing multiple predictors. For the Behavioural Intention equation, comprising Technology Anxiety and Digital Health Literacy, the VIF was 1.77, derived from their inter-construct correlation (*r* = −0.66). For the Perceived Usefulness equation, comprising Perceived Ease of Use and Social Influence, the corresponding VIF was 2.37, derived from their inter-construct correlation (*r* = 0.76). Both VIF values were well below the conservative threshold of 3.3, indicating that multicollinearity was unlikely to bias the structural parameter estimates.

**Table 4:** Retained indicators with standardized factor loadings between 0.50 and 0.69.

Construct	Indicator	Standardized Factor Loading
Perceived Usefulness (PU)	PU1	0.696
Perceived Usefulness (PU)	PU3	0.611
Facilitating Conditions (FC)	FC3	0.561
Technology Anxiety (ANX)	ANX2	0.695
Technology Anxiety (ANX)	ANX3	0.640
Access Barriers (DHB)	DHB1	0.578

### 5.2 Scale descriptive statistics

Item-level means and standard deviations following reverse coding are shown in Table 5. The highest item mean across the entire instrument was PU3 “Digital health services save me time and effort compared with attending the clinic in person” at M = 4.09 (SD = 0.99), with 73.8% of respondents agreeing or strongly agreeing. PU2, concerning the usefulness of AI chatbot health information, returned M = 3.88. These are not trivial endorsements. Participants, on the whole, are not dismissing digital health services as useless they can identify what the tools are for and why they matter.

**Table 5:** Descriptive statistics for likert scale items (*N* = 316).

Construct	Item	Mean	SD
Perceived usefulness	PU1	3.49	1.15
	PU2	3.88	1.06
	PU3	4.09	0.99
	PU4	3.58	1.20
Perceived ease of use	PEOU1	3.37	1.15
	PEOU2	3.63	1.14
	PEOU3(R)	2.35	1.05
Social influence	SI1	3.60	1.19
	SI2	3.58	1.12
	SI3	3.30	1.21
Facilitating conditions	FC1	3.20	1.16
	FC2	3.61	1.17
	FC3	3.34	1.03
	FC4	3.66	1.11
Technology anxiety	ANX1(R)	2.75	1.16
	ANX2	3.77	1.18
	ANX3(R)	2.14	1.31
Behavioural intention	BI1	2.93	1.30
	BI2	2.70	1.25
	BI3(R)	2.57	1.02
Digital health literacy	DHL1	3.77	1.08
	DHL2	3.60	0.99
	DHL3	3.56	1.10
Access barriers	DHB1(R)	2.80	1.04
	DHB2(R)	2.93	1.15
	DHB3(R)	2.35	1.11
Institutional trust	DHT1	3.16	0.89
	DHT2(R)	2.53	0.77

The Behavioural Intention subscale told a very different story. BI1 (intention to continue using Sehhaty) came in at M = 2.93; BI2 (willingness to recommend to peers) at M = 2.70; and BI3 (preference for human clinicians, reverse coded) at M = 2.57. This gap between perceived utility and engagement intention is one of the central puzzles the structural model was designed to address. The access barriers items (DHB1-3, means 2.35–2.93) and anxiety items (ANX1: M = 2.75; ANX3: M = 2.14) indicate that barriers are present and consequential, though not uniformly severe consistent with the moderate IT literacy profile of the sample. Detailed item-level response distributions, including frequencies and percentages for each Likert category, are presented in Supplementary file 1.

### 5.3 Inter-construct correlations

**Figure 2:**
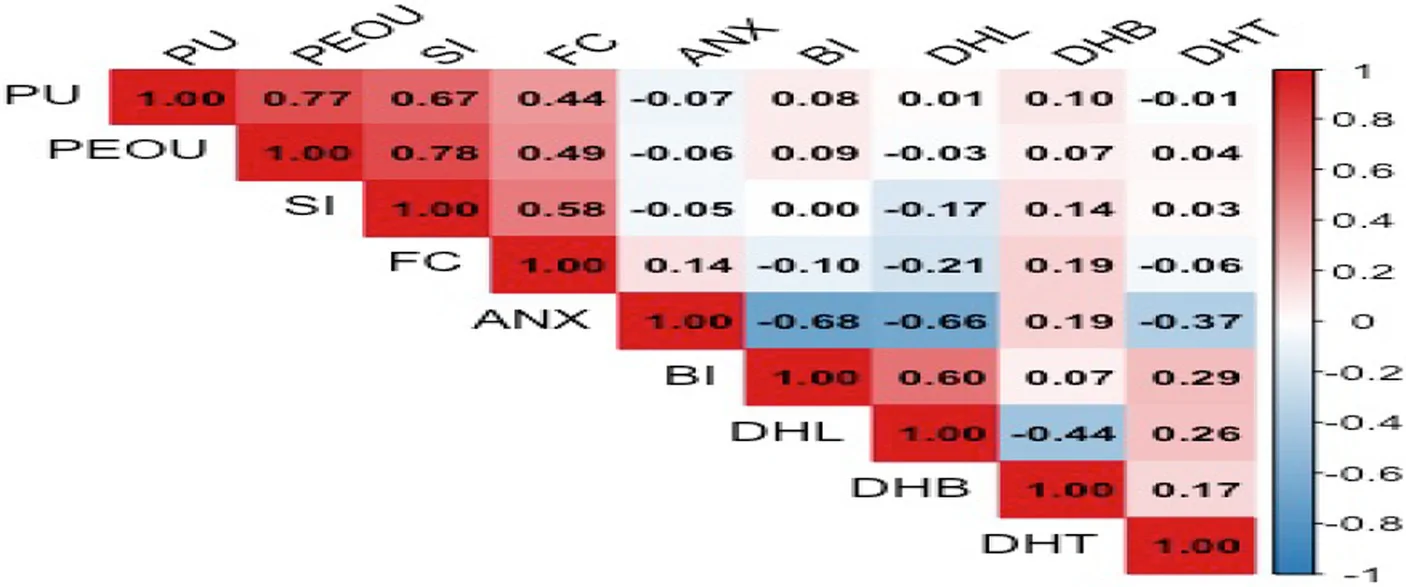
Inter-construct Pearson correlation matrix (*N* = 316). Red shading = positive correlations; blue = negative. Intensity is proportional to magnitude. Most notable inhibitory correlations: ANX↔BI (*r* = −0.68) and ANX↔DHL (*r* = −0.66).

The correlation matrix in Figure 2 is worth examining before moving to the structural results, as it previews several of the most important patterns. Technology anxiety shows the most pervasive inhibitory profile across the matrix it correlates strongly and negatively with both behavioural intention (*r* = −0.68) and digital health literacy (*r* = −0.66), and moderately negatively with institutional trust (*r* = −0.37). The PU-PEOU correlation (*r* = 0.77) and FC-SI correlation (*r* = 0.58) confirms the UTAUT2-theorised relationships between these constructs. One association worth noting specifically is DHB-DHL (*r* = −0.44): access barriers are negatively associated with digital health literacy independently of anxiety, suggesting a direct relationship that the SEM will help disentangle.

### 5.4 Structural equation model

**Figure 3:**
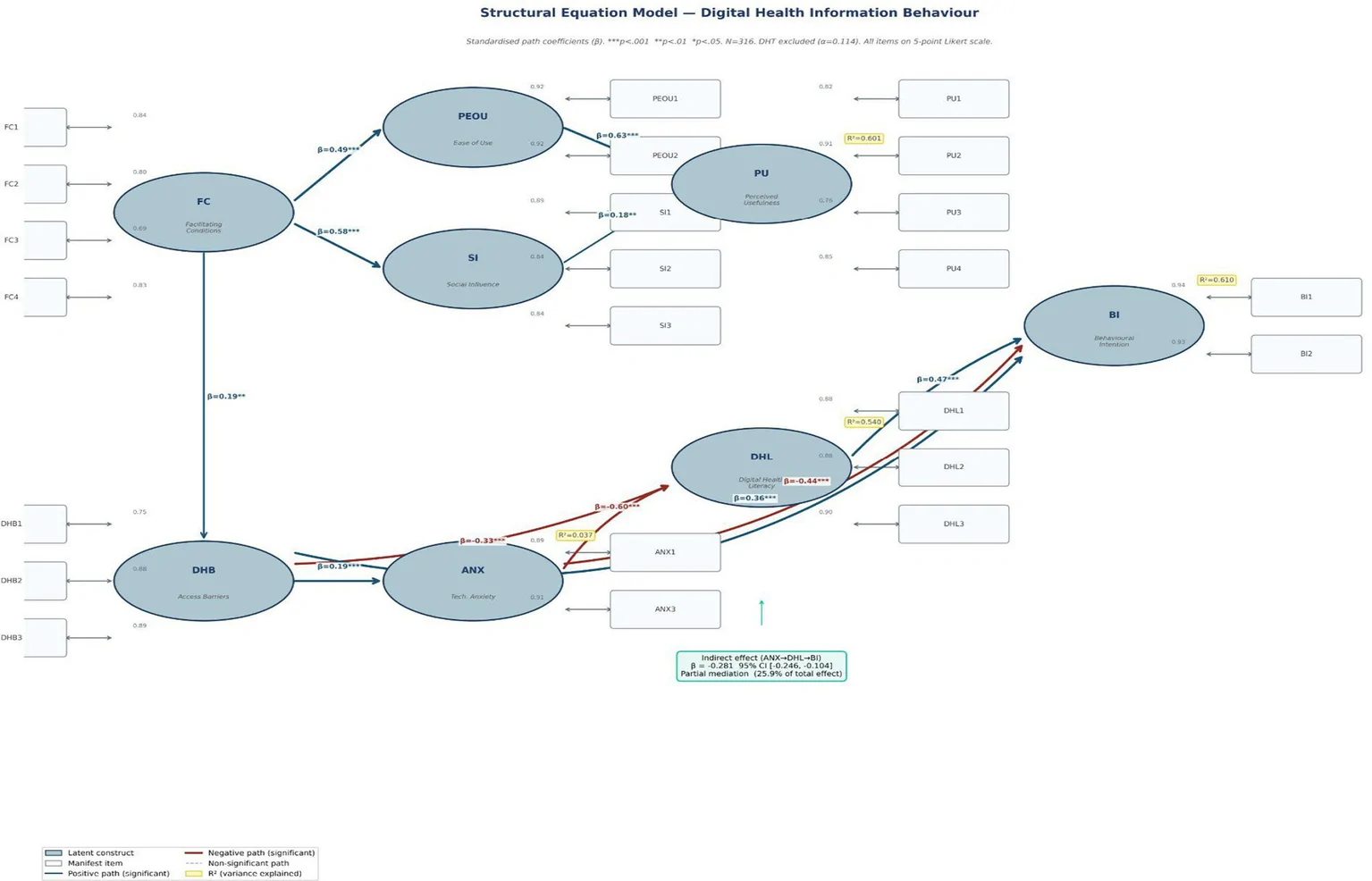
Full SEM path diagram Digital Health Information Behaviour (*N* = 316).

Table 6 reports global fit statistics for the final structural model. The model performed well across all criteria: CFI = 0.947, TLI = 0.938, RMSEA = 0.064 [90% CI: 0.055–0.073], SRMR = 0.071, χ^2^/df = 2.31. All ten hypotheses were supported. The model explained 60.1% of variance in Perceived Usefulness (*R*^2^ = 0.601), 54.0% in Digital Health Literacy (*R*^2^ = 0.540), and 61.0% in Behavioural Intention (*R*^2^ = 0.610). Figure 3 shows the full SEM path diagram with standardised coefficients; Table 7 provides the numerical details with standard errors and hypothesis outcomes.

**Table 6:** Structural equation model global fit indices (*N* = 316).

Fit index	Obtained value	Acceptable threshold	Interpretation
χ^2^/df (CMIN/DF)	2.31	≤3.00	Good fit
CFI	0.947	≥0.90	Good fit
TLI	0.938	≥0.90	Good fit
RMSEA	0.064	≤0.08	Acceptable fit
RMSEA 90% CI	[0.055, 0.073]	Upper bound <0.08	Acceptable fit
SRMR	0.071	≤0.08	Acceptable fit

**Table 7:** SEM standardised path coefficients, standard errors, significance levels, and hypothesis outcomes (*N* = 316).

H	Predictor	Outcome	B	SE	*p*	*R* ^2^	Supported
H1	Facilitating Conditions (FC)	PEOU	0.49	0.062	<0.001		Yes
H2	Facilitating Conditions (FC)	Social Influence (SI)	0.58	0.058	<0.001		Yes
H3	Facilitating Conditions (FC)	Access Barriers (DHB)	-0.19	0.071	0.002		Yes
H4	PEOU	Perceived Usefulness (PU)	0.63	0.054	<0.001	0.601	Yes
H5	Social Influence (SI)	Perceived Usefulness (PU)	0.18	0.065	0.010	0.601	Yes
H6	Access Barriers (DHB)	Technology Anxiety (ANX)	0.33	0.068	<0.001	0.037	Yes
H7	Technology Anxiety (ANX)	Digital Health Literacy (DHL)	-0.60	0.059	<0.001	0.540	Yes
H8	Technology Anxiety (ANX)	Behavioural Intention (BI)	-0.44	0.063	<0.001	0.610	Yes
H9	Digital Health Literacy (DHL)	Behavioural Intention (BI)	0.47	0.061	<0.001	0.610	Yes
H10	ANX → DHL → BI (indirect)	Behavioural Intention (BI)	−0.281		95% CI [−0.246, −0.104]		Yes

Starting from the upstream end of the model: Facilitating Conditions most strongly predicted Social Influence (*B* = 0.58, *p* < 0.001; H2 supported) and Perceived Ease of Use (*B* = 0.49, *p* < 0.001; H1 supported) (see Table 7). This confirms that material infrastructure reliable internet, capable devices, age-appropriate interface design, available technical support is foundational to both how easy older adults find digital health platforms and the social normative environment around their use. FC also negatively predicted Access Barriers (*B* = −0.19, *p* = 0.002; H3 supported), such that participants with better infrastructure reported fewer access difficulties.

The path from PEOU to PU was the largest single coefficient in the model (*B* = 0.63, *p* < 0.001; H4 supported). This has a clear practical implication: improvements to interface navigability produce substantial gains in perceived functional value. Social Influence added a further independent positive effect on PU (*B* = 0.18, *p* = 0.010; H5 supported), confirming that family and clinician endorsement raises perceived usefulness beyond what interface quality alone achieves. Together, these two paths account for 60.1% of variance in PU.

Access Barriers predicted Technology Anxiety (*B* = 0.33, *p* < 0.001; H6 supported). From that point, anxiety produced the two most consequential negative effects in the model: directly suppressing Behavioural Intention (*B* = −0.44, *p* < 0.001; H8 supported) and even more strongly suppressing Digital Health Literacy (*B* = −0.60, *p* < 0.001; H7 supported). Literacy, in turn, was a strong positive predictor of Intention (*B* = 0.47, *p* < 0.001; H9 supported). The overall picture is one of a barrier cascade: physical and experiential access difficulties translate into anxiety, which then simultaneously discourages direct engagement and erodes the literacy competencies that would otherwise support it.

### 5.5 Mediation analysis

The bootstrapped mediation test (5,000 replications) confirmed a statistically significant indirect effect of Technology Anxiety on Behavioural Intention through Digital Health Literacy: indirect *B* = −0.281 (95% CI [−0.246, −0.104]; H10 supported). This indirect effect accounts for 25.9% of the total anxiety-intention effect, constituting partial mediation. The direct effect of anxiety on intention remained significant (*B* = −0.44, *p* < 0.001) after including the mediator, indicating that anxiety operates through two distinct routes: an affective pathway in which apprehension directly discourages engagement, and a cognitive-developmental pathway in which anxiety suppresses the literacy skills that would otherwise make confident platform use achievable. Any intervention targeting only one of these routes is likely to produce incomplete results.

### 5.6 Non-parametric group comparisons

**Figure 4:**
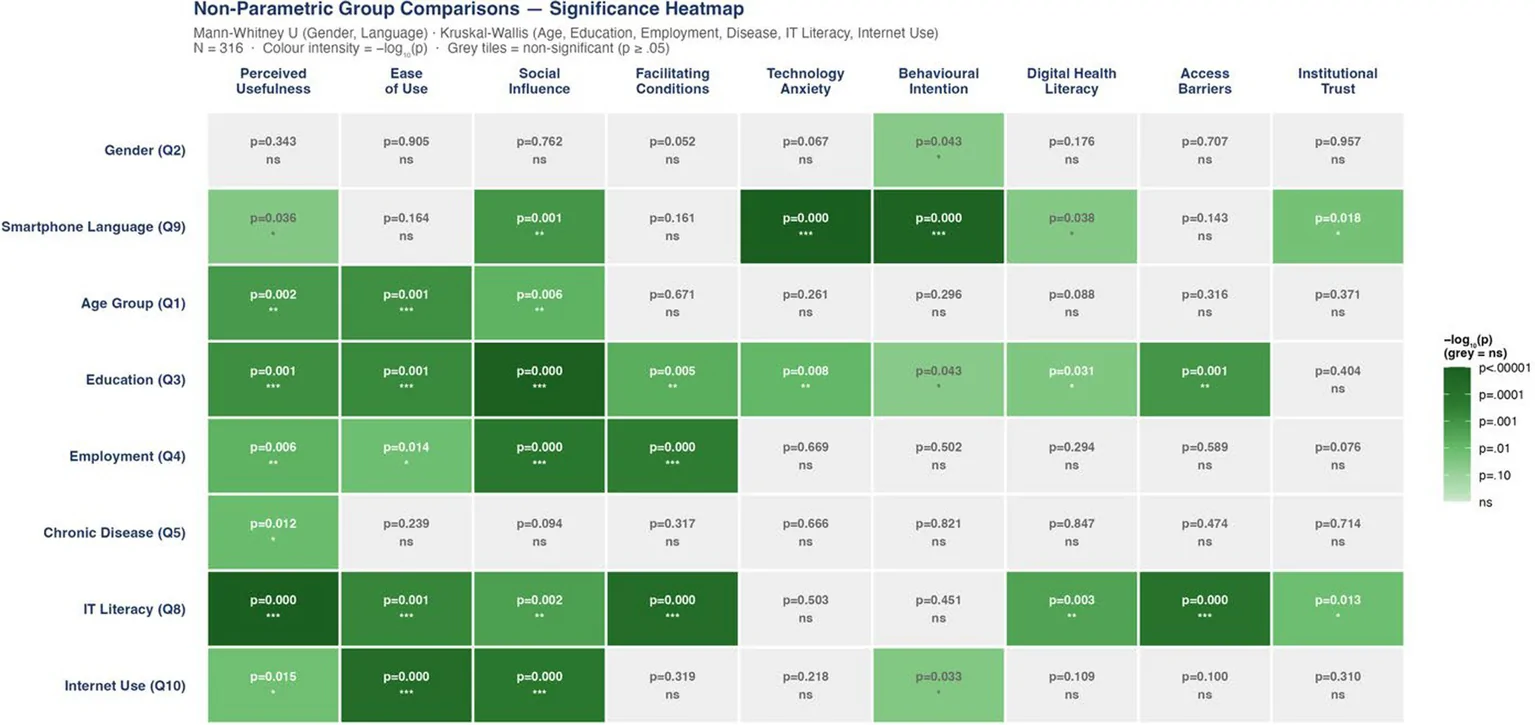
Non-parametric group comparisons significance heatmap (*N* = 316).

Figure 4 shows the significance heatmap for group comparisons across all eight constructs and eight sociodemographic variables. Smartphone language, education level, and IT literacy each showed significant associations across five or more constructs, making them the sociodemographic variables with the broadest discriminative reach in this dataset. Gender and chronic disease status were largely non-significant within this educationally advantaged, digitally exposed sample, neither variable meaningfully differentiated digital health access profiles. That finding should not be extrapolated to the broader population, but it does suggest that within samples of this type, intervention targeting by gender is unlikely to be the most efficient strategy.

**Figure 5:**
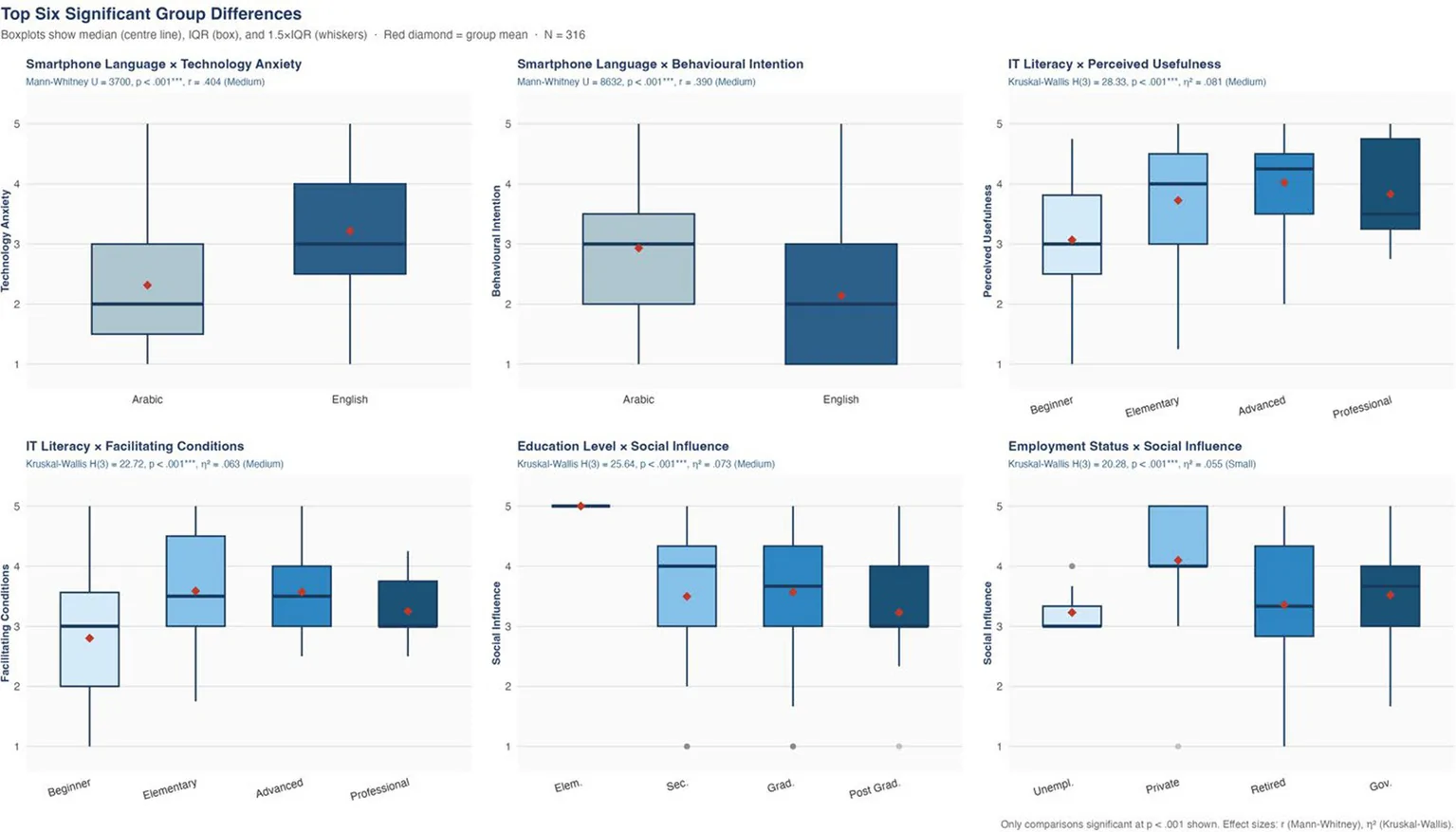
Six largest significant group differences (*N* = 316).

Figure 5 shows the six largest significant pairwise differences as boxplots. The most striking result is the smartphone language comparison for Technology Anxiety: Arabic users reported substantially lower anxiety than English users (*U* = 3,700, *p* < 0.001, *r* = 0.404, medium effect), with the Arabic group’s median score approximately one full scale point lower. This runs counter to the intuitive expectation that people with stronger digital skills more likely to use English-language interfaces would also feel less anxious about health platforms. What the data suggest instead is that native language interface use reduces the cognitive and interpretive demands that generate anxiety in health-specific contexts. Reading a test result, completing a registration form, understanding a medication instruction all of these tasks require meaningfully less effort in Arabic for this population. The further finding that Arabic users also showed significantly lower behavioural intention (*U* = 8,632, *p* < 0.001, *r* = 0.390) indicates that reduced anxiety does not automatically translate into stronger engagement intent, pointing to additional barriers that operate independently of anxiety for this group.

Among IT literacy comparisons, PU scores rose steadily from beginner to advanced level (H(3) = 28.33, *p* < 0.001, η^2^ = 0.081), with a similar though slightly flatter gradient for Facilitating Conditions (H(3) = 22.72, *p* < 0.001, η^2^ = 0.063). For education level, the social influence comparison was notable: elementary-education participants showed a higher median and greater variability (H(3) = 25.64, *p* < 0.001, η^2^ = 0.073), consistent with less stable social endorsement networks around technology use. Employment status showed a small but significant effect on Social Influence (H(3) = 20.28, *p* < 0.001, η^2^ = 0.055), with unemployed participants reporting the lowest social endorsement consistent with reduced exposure to workplace and peer digital health norms (see Table 8).

**Table 8:** Key significant non-parametric group comparisons across construct domains (*N* = 316).

Variable	Construct	Test	Statistic	Effect
Smartphone language	Technology anxiety	Mann–Whitney U	U = 3,700, *p* < 0.001	*r* = 0.404
Smartphone language	Behavioural intention	Mann–Whitney U	U = 8,632, *p* < 0.001	*r* = 0.390
Smartphone language	Digital health literacy	Mann–Whitney U	*p* = 0.038	*r* = 0.118 (small)
Smartphone language	Social influence	Mann–Whitney U	*p* = 0.001	*r* = 0.182 (small-medium)
IT literacy	Perceived usefulness	Kruskal-Wallis	H(3) = 28.33, *p* < 0.001	η^2^ = 0.081
IT literacy	Facilitating conditions	Kruskal-Wallis	H(3) = 22.72, *p* < 0.001	η^2^ = 0.063
IT literacy	Access barriers	Kruskal-Wallis	H(3) = 19.47, *p* < 0.001	η^2^ = 0.058
Education level	Social influence	Kruskal-Wallis	H(3) = 25.64, *p* < 0.001	η^2^ = 0.073
Age group	Perceived usefulness	Kruskal-Wallis	H(3) = 14.82, *p* = 0.002	η^2^ = 0.041
Age group	Ease of use	Kruskal-Wallis	H(3) = 16.54, *p* = 0.001	η^2^ = 0.047
Employment status	Social influence	Kruskal-Wallis	H(3) = 20.28, *p* < 0.001	η^2^ = 0.055

### 5.7 Health information behaviour

**Figure 6:**
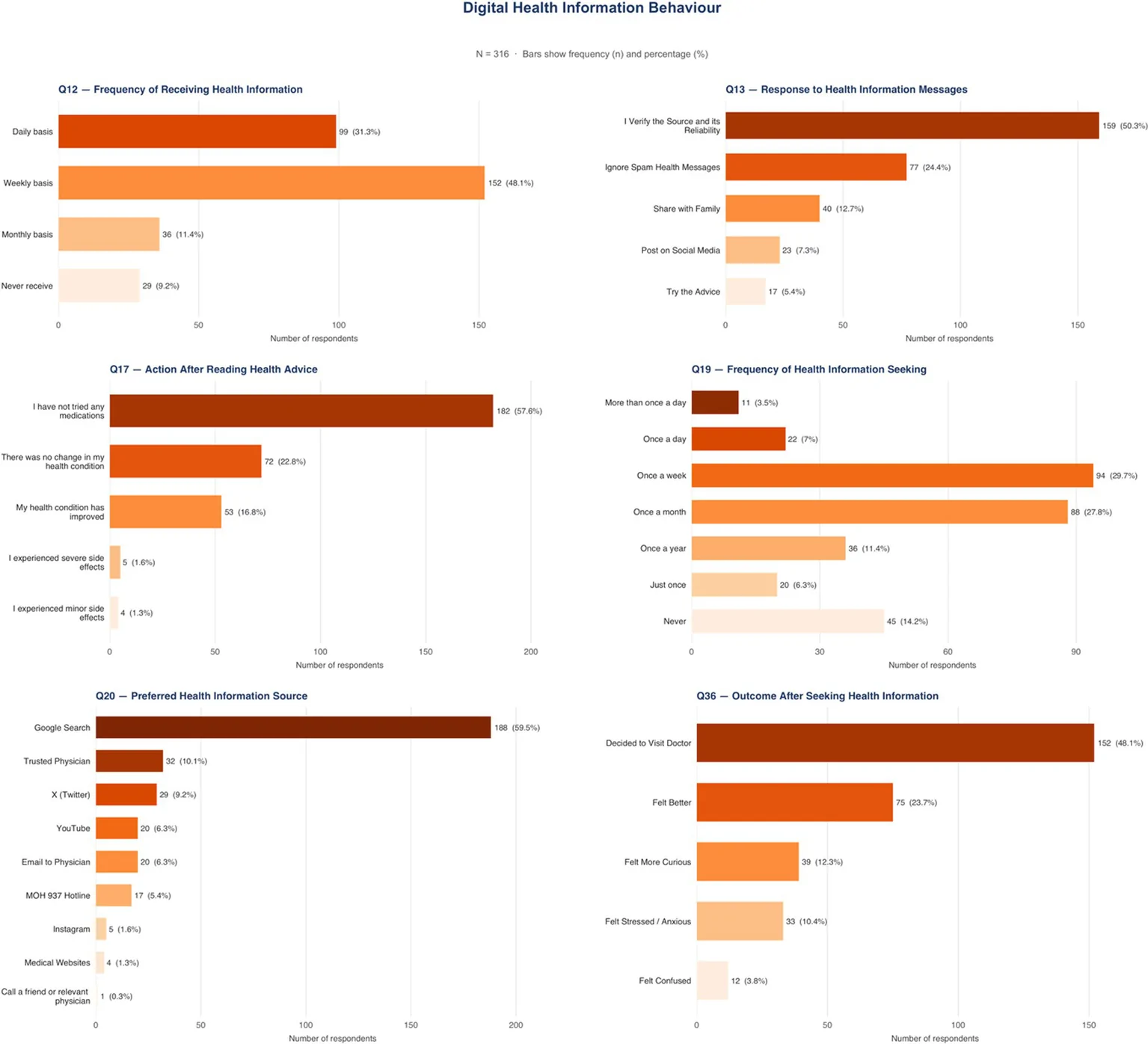
Digital health information behaviour (*N* = 316).

Figure 6 summarises health information behaviour across six survey items. Google was the dominant source of health information (59.5%), substantially ahead of trusted physician consultation (10.1%) and official MOH channels (5.4%). Notably, Google was also the most common verification method for health information (*n* = 214 participants), which means a substantial portion of the sample is using the same search engine as both primary and secondary information source a circular appraisal pattern that offers limited epistemic protection. Source and reliability verification was the most common response to receiving health messages (50.3%), suggesting that critical appraisal behaviour is present, even if constrained by tool dependency. The most common downstream outcome of health information seeking was deciding to visit a physician (48.1%) a reminder that digital health information in this group is functioning primarily as a gateway to formal care, not a substitute for it.

**Figure 7:**
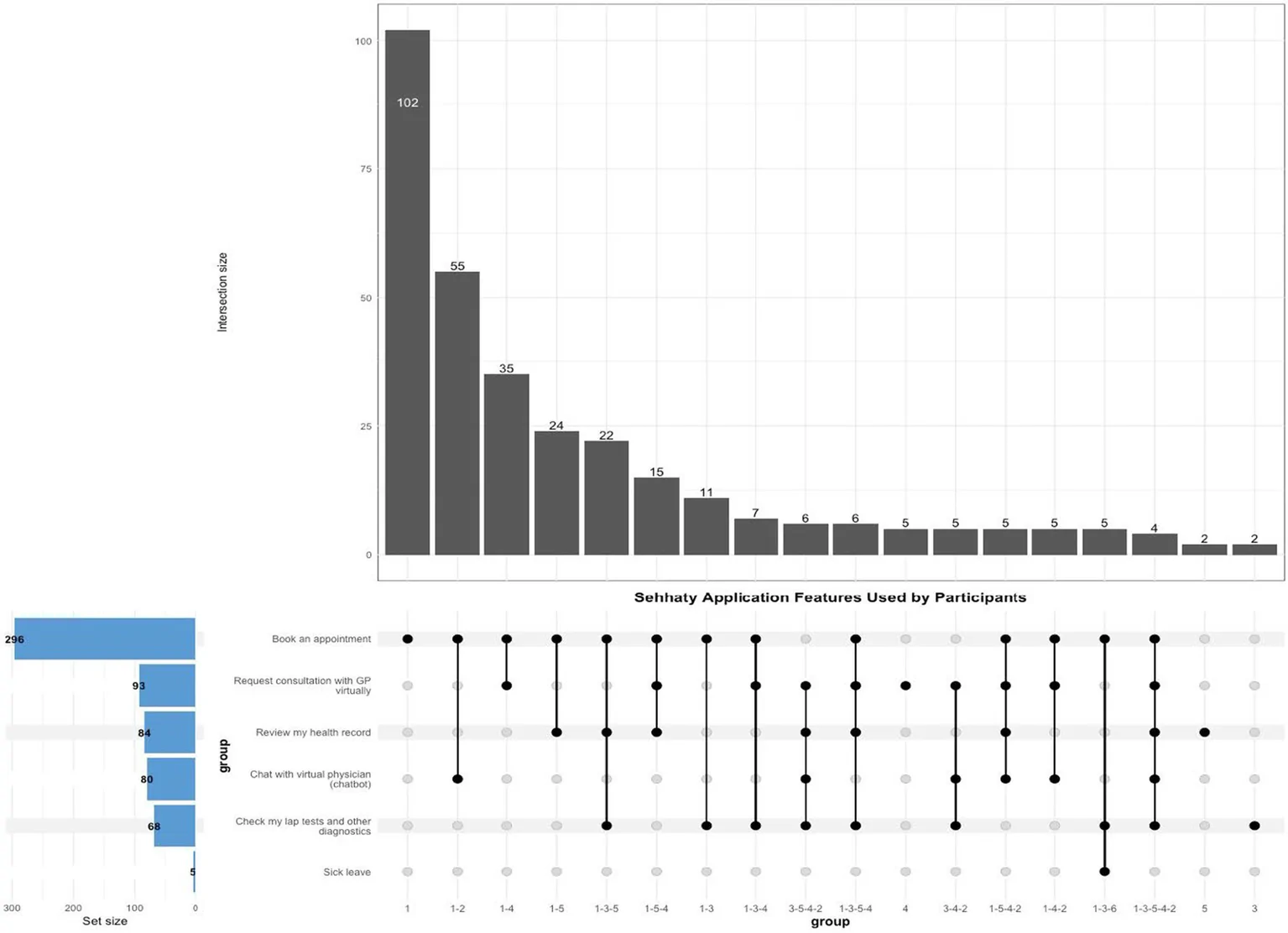
Sehhaty application features used by participants.

Figure 7 presents the UpSet plot of Sehhaty feature combinations used by participants. Appointment booking was used by 296 participants effectively the entire sample while every other feature dropped off sharply: virtual GP consultation (*n* = 93), health record review (*n* = 84), chatbot interaction (*n* = 80), and diagnostic results review (*n* = 68). The most common single-feature profile was appointment booking alone (*n* = 102), and multi-feature combinations involving higher-order functions were comparatively rare. This tiered engagement pattern is not coincidental it maps directly onto the anxiety-literacy cascade identified in the structural model. The features that participants avoided are precisely those requiring interpretive judgement under conditions of perceived risk: reading a laboratory result, following a chatbot recommendation, reviewing diagnostic data. These are the tasks that ANX3 worry about making a health-consequential mistake speaks to most directly. Sehhaty is, in practice, functioning as an appointment booking system for most older adults in this sample, with its clinical intelligence features largely untouched. That is not a failure of awareness but a predictable consequence of unaddressed anxiety and literacy barriers, and it represents a substantial unrealised potential for the platform’s contribution to chronic disease self-management in this population.

**Figure 8:**
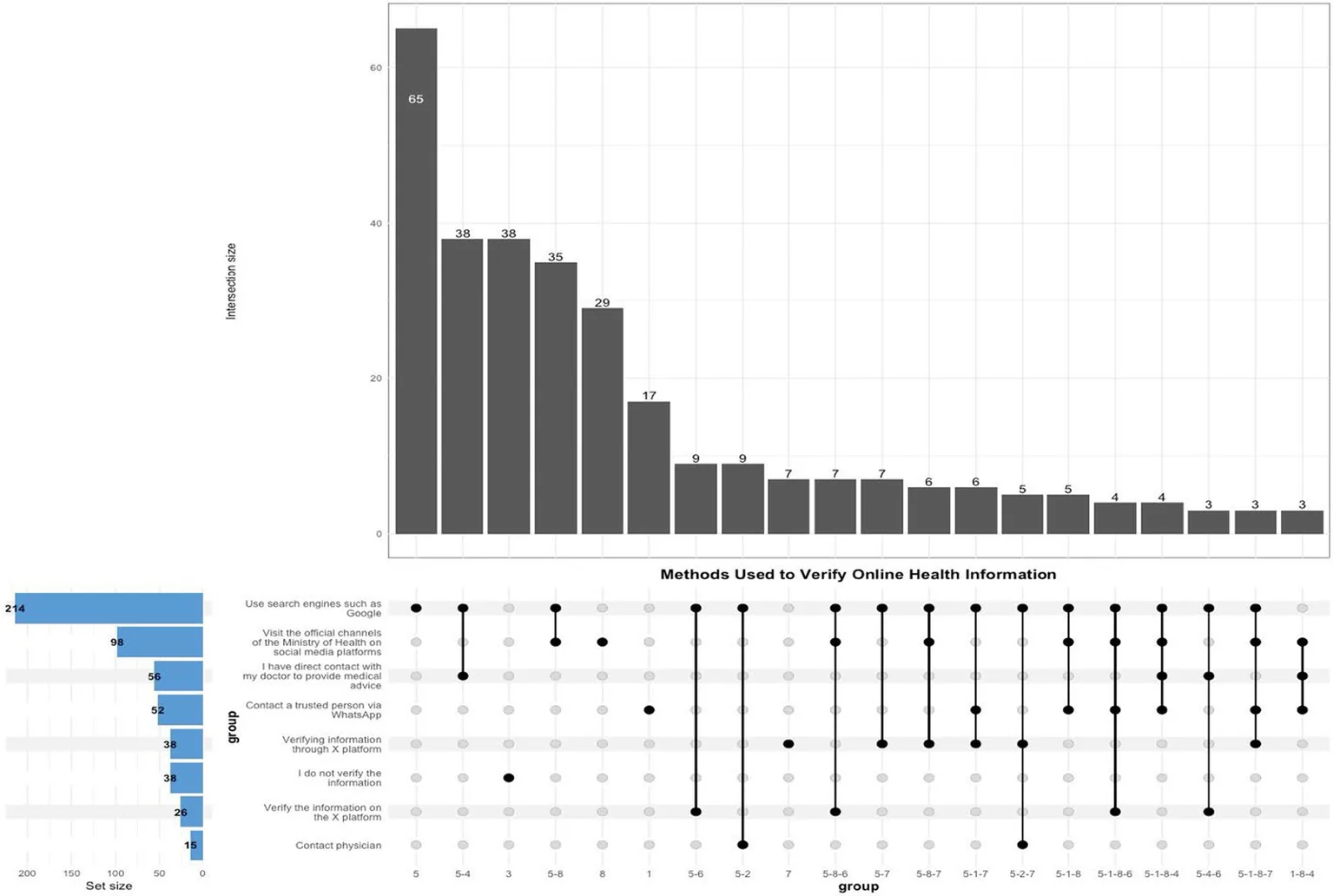
Methods used to verify online health information.

Figure 8 documents how participants verified the health information they received via texts or social media. Google Search was the most widely used verification method (*n* = 214), followed by visiting official Ministry of Health channels (*n* = 98), direct physician contact for verification (*n* = 56), and consulting a trusted person via WhatsApp (*n* = 52). The most common single-method profile was Google alone (*n* = 65). The clinical significance of this pattern lies in its circularity: Google was identified in Section 3.9 as the dominant primary source of health information, and it is also the most common verification tool. A substantial proportion of participants are, in effect, using the same unmoderated search engine to both find and confirm health information a practice that offers no genuine epistemic protection against inaccurate or misleading content. Formal verification through official MOH channels, which would represent meaningful quality assurance, reached fewer than a third of participants. The verification behaviour data therefore qualify the otherwise encouraging finding that 50.3% of participants said they verified health information sources: the verification is happening, but largely through a mechanism that does not reliably distinguish accurate health information from the herbal remedy and self-medication content that dominates the informal information channels documented in Figures 3 and 4. Strengthening digital health literacy in this population must include explicit attention to source hierarchy and verification method quality, not only the habit of verification itself.

**Figure 9:**
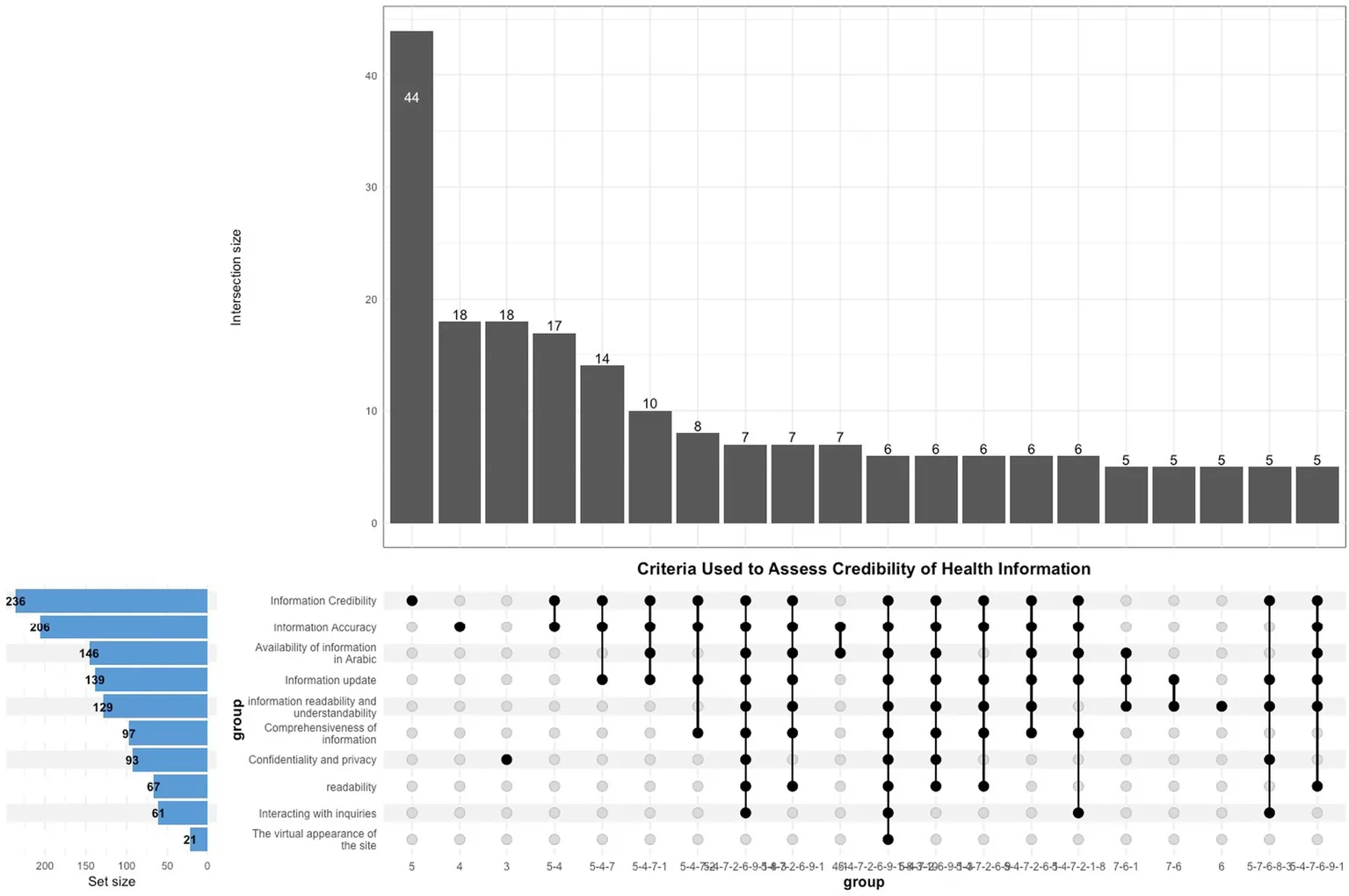
Criteria used to assess credibility of health information.

Figure 9 reveals the criteria participants applied when judging the credibility of digital health information. Information credibility ranked first (*n* = 236) and accuracy second (*n* = 206) both expected primary epistemic standards. What stands out is the third-ranked criterion: availability of information in Arabic (*n* = 146), which ranked ahead of information recency (*n* = 139), readability (*n* = 129), and comprehensiveness (*n* = 97). This is not a minor finding. It provides independent empirical corroboration from a completely different analytical plane for the language-anxiety interaction identified in the non-parametric comparisons. Language availability is not functioning as a usability preference for this population; it is operating as a credibility criterion. Information that is not in Arabic is harder to evaluate, harder to trust, and harder to act on with confidence, regardless of its clinical quality. Confidentiality and privacy concerns ranked seventh (*n* = 93), suggesting that while privacy is present in participants’ credibility calculus, it is considerably less determinative than language and accuracy. Taken together, these data make a straightforward argument: for Arabic-speaking older adults, the epistemic accessibility of digital health information is inseparable from its linguistic accessibility. Platform design and health communication strategies that treat Arabic-language content as a translation exercise rather than a foundational trust requirement are misreading what this population actually needs.

## 6 Discussion

The headline finding is clear: technology anxiety is the dominant structural inhibitor of digital health engagement in this sample of older Saudi adults, operating through two pathways directly suppressing behavioural intention and indirectly through its erosion of digital health literacy. An *R*^2^ of 0.61 for behavioural intention is notably higher than the 0.30–0.55 range typical in mHealth adoption SEM studies (11, 30), and all ten hypothesised structural paths were supported. Several findings within the model, however, warrant more detailed interpretation.

The dual-pathway mediation result is arguably the most theoretically significant contribution of this study. Song, Yang (31) in their 2025 systematic review of mHealth adoption among older adults identified inadequate technical literacy as one of the most consistently reported adoption barriers, alongside functional limitations and technology-related challenges (31) but their review, like most in this area, did not model the structural mechanism through which anxiety suppresses literacy development. The present mediation analysis provides that mechanism: the indirect effect through digital health literacy accounts for approximately a quarter of anxiety’s total negative influence on intention, and the direct path remains substantial after the mediator is included. Eysenck et al.’s attentional control theory offers a plausible explanation anxiety depletes cognitive resources available for acquiring and processing new information, which would slow literacy skill development alongside its more immediate motivational effects. If this explanation holds, then interventions targeting literacy alone will be of limited value for the most anxious users, because anxiety is actively interfering with the learning process itself. The affective and cognitive dimensions need to be addressed together.

The 2024 scoping review by Shi and colleagues identified education level, social support from family members, and IT literacy as the most consistently reported determinants of digital health literacy among older adults (32) findings that are directly consistent with the group comparison results in the present study, where education level (H(3) = 25.64, *p* < 0.001) and IT literacy (H(3) = 28.33, *p* < 0.001) both showed significant associations with DHL scores. Importantly, Shi, Du (32) also found that limited English proficiency significantly restricted access to eHealth resources among older migrants, affecting their digital health literacy (32) a finding that resonates with the language-anxiety interaction documented here, though from a different analytical angle. The implication in both cases is the same: language is not a peripheral variable in digital health literacy research but a structural one.

The finding that Perceived Ease of Use accounts for 60.1% of variance in Perceived Usefulness (*B* = 0.63, *p* < 0.001) the strongest single path in the model warrants specific attention. A 2025 systematic scoping review on digital health acceptance for non-communicable disease management found that perceived usefulness, ease of use, and social influence positively impact older adults’ acceptance, while usability issues, technical challenges, and privacy concerns persist as barriers that must be addressed to promote long-term adoption (33). The present data extend this further: ease of use is not merely a parallel adoption driver alongside usefulness it is the prerequisite through which usefulness perceptions are formed. The 2025 systematic review on barriers and facilitators of digital health adoption among older adults with chronic diseases similarly identified user-friendly design and training as the primary capability facilitators, with trusted clinician endorsement and perceived benefits of digital health as key motivational drivers (17). These findings further suggest that interface redesign and literacy training are not competing intervention priorities but sequential ones ease of use must be established before perceived usefulness can drive intention.

The Facilitating Conditions findings confirm that infrastructure is not a background variable but an active structural lever. Its effects on engagement intention are distributed across multiple pathways through ease of use, through social normative environments, and through the barrier-anxiety cascade. Barriers related to poor internet access, high costs, and lack of integrated technical support were among the most prominently identified structural barriers in the 2025 JMIR Ageing systematic review (17), consistent with the present FC findings where internet connectivity (FC1; M = 3.20) and interface age-appropriateness (FC3; M = 3.34) were the lowest-scoring items within the construct. Song, Yang (31) review similarly identified lack of critical resources and external infrastructure constraints as the primary adoption barriers operating across patient, caregiver, and provider stakeholder groups (31), suggesting that infrastructure dependency is a generalisable feature of older adult digital health engagement rather than one specific to any particular context.

The language-anxiety interaction is among the most novel findings of this study and, to the best of this author’s knowledge, has no direct precedent in the published mHealth or digital health literacy literature. The effect size (*r* = 0.404, medium) is large enough to be practically meaningful. This outcome is contrary to the intuitive expectation that more digitally experienced users would feel less anxious. A possible explanation is that navigating health-specific content in a non-native language generates anxiety tied specifically to the consequentiality of health decisions rather than to general technological unfamiliarity. Alfaris, Alhazzani (34) found that 86% of health-related WhatsApp messages circulating in Saudi Arabia contained invalid or inaccurate content, with 83.7% originating from unknown sources (34) a finding that reinforces why Arabic language availability functions as a credibility criterion rather than merely a usability preference in this population. When health information quality is this variable, the ability to evaluate content in one’s first language becomes epistemically protective. The convergent finding that Arabic availability ranked third among health information credibility criteria (*n* = 146, Figure 9), ahead of information recency, provides independent corroboration from a different analytical plane: language is tied to whether information feels trustworthy and actionable, not simply whether it is convenient to read.

The tiered Sehhaty usage profile appointment booking used by 296 participants, chatbot interaction by only 80 provides what might be described as ecological validity for the structural findings. The features older adults avoid are precisely those requiring interpretive judgement under conditions of perceived risk. The 2025 scoping review on digital health acceptance for NCD management noted that although older adults generally hold positive attitudes toward digital health tools, barriers related to usability and technical complexity persist and must be addressed to promote deeper engagement beyond basic transactional use (33). This is consistent with the risk-minimisation interpretation proposed here and corroborates Wildenbos, Peute (14) and Knowles and Hanson (35) qualitative findings. The convergence across structural modelling and real-world usage data lends greater confidence to the interpretation than either source of evidence alone would support.

WhatsApp’s dominance across health information reception, physician contact, and source verification channels raises concerns that extend beyond technology adoption metrics. Alfaris, Alhazzani (34) demonstrated that WhatsApp is the most frequently used social media platform in Saudi Arabia (89.9% of internet users), with 34.5% using it to search for health-related information yet the same study found that the great majority of health messages circulating through the platform were invalid or inaccurate (34). A subsequent intervention study by Alsaad and AlDossary (36) found that educational video interventions could significantly improve health misinformation identification on WhatsApp among Saudi populations (36), suggesting that literacy-focused channel-specific interventions are both needed and feasible. This population has effectively constructed a parallel informal health communication infrastructure that bypasses formal digital health platforms for a large proportion of everyday interactions. Shi et al. (32)’s scoping review further highlighted that the frequency of receiving guidance from family members significantly affected digital health literacy in older adults a finding that aligns with the Social Influence → Perceived Usefulness path in the present model (*B* = 0.18, *p* = 0.010) and suggests that family networks embedded within WhatsApp communication channels represent an underutilised vehicle for digital health literacy promotion. Strengthening DHL in this population is therefore not only about improving platform adoption metrics; it has direct implications for how older adults evaluate the informal health content that reaches them daily through channels the formal health system does not control.

More broadly, these patterns situate the findings within the growing field of health misinformation and disinformation research. The circulation of unverified, and at times deliberately misleading, health content through informal channels constitutes an infodemic risk that disproportionately affects older adults, who combine high exposure with a lower capacity for critical appraisal. On this reading, digital health literacy is not only an access-and-use competency but a frontline defence against health misinformation, since the skills of locating, appraising, and interpreting online health information are precisely those required to resist it (15, 16). Future research should therefore examine, using longitudinal and experimental designs, whether targeted digital-health-literacy interventions build measurable misinformation resilience in older Arabic-speaking populations, and how trusted national platforms such as Sehhaty might be positioned as authoritative counterweights to the informal information ecosystem aligning the practical recommendations of this study with the wider imperative of infodemic management.

Several limitations should be acknowledged. The cross-sectional design cannot establish the causal direction of the anxiety-literacy relationship. Whilst the anxiety → literacy suppression pathway is theoretically well-supported by attentional control theory, the reverse direction low literacy generating greater platform anxiety is equally plausible and cannot be ruled out. Longitudinal designs are needed to test directionality over time. The sample’s educational profile (82.9% graduate or above) and prior-platform-use eligibility criterion mean that barrier and anxiety scores are almost certainly conservative estimates relative to the broader Saudi older adult population. The 2025 updated systematic review on digital health adoption barriers specifically noted that most existing studies focus on general populations or specific diseases, with limited attention to older adults from lower socioeconomic or rural backgrounds a gap the present study’s sampling strategy likely perpetuates (17). The exclusion of Institutional Trust from the structural model, while empirically justified, leaves privacy concerns analytically undertreated. Self-report measures of anxiety and platform use carry inherent social desirability risk. Finally, the specificity of the Saudi Arabian context means that findings should not be transferred to other GCC or Arabic-speaking national contexts without independent replication.

## 7 Theoretical and practical implications

### 7.1 Theoretical implications

This study advances acceptance theory in three respects. First, it reframes technology anxiety and digital health literacy from parallel predictors into an ordered mechanism. By specifying and finding support for an access-barrier → anxiety → literacy → intention cascade, with a significant indirect pathway accounting for roughly a quarter of anxiety’s total association with intention, the study moves the field from cataloguing correlates of older-adult non-engagement toward modelling the process that connects them. This responds to a recurring critique that acceptance models leave older-adult variance unexplained because they omit the interlocking affective and cognitive barriers specific to this group (32).

Second, it positions anxiety as an upstream driver of literacy rather than a co-equal correlate. Interpreted through attentional control theory, the anxiety → literacy path implies that anxiety may interfere with the very learning process by which literacy develops. If so, literacy-only interventions will be of limited value for the most anxious users, and models that enter anxiety and literacy as independent predictors will misattribute effects that are in fact sequentially linked (13).

Third, it identifies language concordance as a theoretically meaningful and previously unmodelled determinant of anxiety. That Arabic-interface users reported substantially lower anxiety than English-interface users despite lower average proficiency corroborated independently by the ranking of Arabic availability as a credibility criterion suggests that for health-consequential tasks, language operates not as a usability preference but as an epistemic-trust variable. This extends acceptance theory by indicating that effort expectancy and anxiety in multilingual health systems are partly a function of linguistic, not merely technical, accessibility (15, 16).

### 7.2 Practical implications

The findings translate into four prioritised recommendations, ordered by the strength of their supporting evidence in the model.

*Sequence usability redesign ahead of feature promotion*. Because ease of use is the largest path to usefulness in the model and the usage data show systematic avoidance of interpretively demanding features, age-appropriate usability redesign of Sehhaty is the highest-leverage intervention: ease of use is the prerequisite through which usefulness perceptions, and ultimately intention, are formed.

*Treat Arabic-language-first design as a patient-safety decision, not a localisation convenience*. Native-language concordance reduces anxiety with a medium effect and ranks among the top credibility criteria; interfaces, result explanations, and chatbot interactions should be designed in Arabic first.

*Design literacy programmes around source criticality and result interpretation, not basic access*. The primary literacy challenge in this population is evaluating the informal, often WhatsApp-borne, health content already saturating their networks; community programmes should target verification-method quality and the interpretation of results and recommendations (17).

*Embed clinician endorsement into routine chronic-disease consultations*. Social influence raises perceived usefulness independently of interface quality, and family and clinician networks are an under-used vehicle for literacy promotion; structured clinician endorsement is a low-cost amplifier of engagement (32).

Crucially, the indirect-pathway finding implies these levers are complementary rather than substitutable: addressing anxiety without literacy, or literacy without anxiety, risks leaving the cascade intact. Interventions are likely to be most effective when affective, competence-related, and structural barriers are addressed together.

## 8 Conclusion

The gap between older Saudi adults’ positive perceptions of digital health services and their low engagement intention is driven by technology anxiety and digital health literacy deficits operating through structurally linked mechanisms. The partial mediation of anxiety’s effect through literacy 25.9% of the total effect establishes the clearest intervention signal: addressing these constructs in combination is necessary because each independently sustains the other’s limiting effects. Four recommendations follow, in order of the strength of their supporting evidence. The Sehhaty platform requires age-appropriate usability redesign as the highest priority because PEOU → PU (*B* = 0.63) is the largest path in the model and the usage data confirm that complex features are systematically avoided. Arabic-language-first design should be treated as a patient safety decision, given that language concordance reduces anxiety with a medium effect and ranks third among health information credibility criteria. Community-based digital health literacy programmes should prioritise source criticality and result interpretation skills, recognising that the primary literacy challenge in this population is not accessing health information but critically evaluating the informal content already saturating their social networks. Finally, clinician endorsement of Sehhaty should be integrated into routine chronic disease consultations, given its demonstrable amplifying effect on perceived usefulness. Further research using longitudinal designs and probability samples is needed to confirm the directionality of the anxiety-literacy pathway and to establish the generalisability of these findings across GCC contexts.

## Data Availability

The raw data supporting the conclusions of this article will be made available by the authors, without undue reservation.
