# Supplementary material for: Technology anxiety and digital health literacy as structural determinants of mHealth engagement among older adults: a SEM study in Saudi Arabia
Source: Front Public Health. 2026 Aug 14;14:1872211. doi: 10.3389/fpubh.2026.1872211 (PMC13522229; doi:10.3389/fpubh.2026.1872211)
Supplement: Supplementary file 1 [file Data_Sheet_1.PDF]

Supplementary file 1

Item	Item Wording	N	Mean	SD	Strongly Disagree (1)		Disagree (2)		Neutral (3)		Agree (4)		Strongly Agree (5)	
					n	%	n	%	n	%	n	%	n	%
**Perceived Usefulness**														
**PU1**	Using digital health services such as Sehhaty helps me manage my chronic condition more effectively without needing to visit the hospital.	316	3.49	1.15	19	6.0	41	13.0	93	29.4	92	29.1	71	22.5
**PU2**	AI-enabled health tools such as virtual physician chatbots provide me with useful and reliable information for managing my health.	316	3.88	1.06	8	2.5	33	10.4	51	16.1	122	38.6	102	32.3
**PU3**	Digital health services save me time and effort compared with attending the clinic in person.	316	4.09	0.99	7	2.2	11	3.5	65	20.6	96	30.4	137	43.4
**PU4**	Using digital health services reduces the number of unnecessary hospital or clinic visits I make.	316	3.58	1.20	17	5.4	48	15.2	76	24.1	85	26.9	90	28.5
**Perceived Ease of Use**														
**PEOU1**	I find it easy to navigate the Sehhaty application to complete tasks such as booking appointments or reviewing my test results.	316	3.37	1.15	22	7.0	55	17.4	73	23.1	115	36.4	51	16.1

**PEOU2**	The language and instructions used in digital health applications are clear enough for me to use them without assistance from others.	316	3.63	1.14	16	5.1	30	9.5	99	31.3	80	25.3	91	28.8
**PEOU3 (R)**	*Age-related difficulties, such as reading small text or pressing small buttons, make it hard for me to use digital health applications.*	316	2.35	1.05	72	22.8	111	35.1	96	30.4	23	7.3	14	4.4
**Social Influence**														
**SI1**	My family members or close friends encouraged me to use digital health services such as Sehhaty or virtual physician chatbots.	316	3.60	1.19	23	7.3	30	9.5	85	26.9	91	28.8	87	27.5
**SI2**	My physician or healthcare provider recommended that I use digital health applications to manage my condition.	316	3.58	1.12	17	5.4	31	9.8	99	31.3	91	28.8	78	24.7
**SI3**	People whose opinions I value believe that using digital health services is beneficial for managing my health.	314	3.30	1.21	34	10.8	27	8.6	129	41.1	58	18.5	66	21.0
**Facilitating Conditions**														
**FC1**	I have access to a reliable internet connection that allows me to use digital health services whenever I need them.	314	3.20	1.16	26	8.3	53	16.9	121	38.5	59	18.8	55	17.5
**FC2**	I receive sufficient technical support from	309	3.61	1.17	19	6.1	30	9.7	91	29.4	80	25.9	89	28.8

	family members or healthcare staff when I encounter difficulties using digital health applications.													
**FC3**	The physical design of digital health applications, including text size, button size, and screen layout, is suitable for someone my age.	316	3.34	1.03	20	6.3	27	8.5	138	43.7	87	27.5	44	13.9
**FC4**	I own a device such as a smartphone or tablet that is capable of running digital health applications effectively.	309	3.66	1.11	16	5.2	25	8.1	89	28.8	98	31.7	81	26.2
**Technology Anxiety**														
**ANX1 (R)**	*I feel anxious or uncomfortable when I am required to use a new digital health application for the first time.*	316	2.75	1.16	48	15.2	97	30.7	74	23.4	79	25.0	18	5.7
**ANX2**	I feel confident and at ease when using digital health tools to look after my health.	316	3.77	1.18	0	0.0	66	20.9	64	20.3	62	19.6	124	39.2
**ANX3 (R)**	*I am worried that I might make a serious mistake when using a digital health application that could affect my health.*	316	2.14	1.31	144	45.6	65	20.6	52	16.5	29	9.2	26	8.2
**Behavioural Intention**														
**BI1**	I intend to continue using digital health services such as Sehhaty and virtual physician chatbots to	316	2.93	1.30	54	17.1	58	18.4	118	37.3	28	8.9	58	18.4

	support my healthcare in the future.												
**BI2**	I would recommend digital health services such as Sehhaty to other older adults managing chronic conditions.	316	2.70	1.25	64	20.3	74	23.4	113	35.8	22	7.0	43	13.6
**BI3 (R)**	*I prefer speaking with a healthcare professional rather than relying solely on digital health services.*	316	2.57	1.02	62	19.6	67	21.2	140	44.3	39	12.3	8	2.5
	**Digital Health Literacy**													
**DHL1**	I feel comfortable searching for health information using digital platforms such as websites or mobile applications.	314	3.77	1.08	8	2.5	27	8.6	97	30.9	78	24.8	104	33.1
**DHL2**	I understand how to interpret the results provided by digital health self-assessment tools.	316	3.60	0.99	0	0.0	30	9.5	152	48.1	48	15.2	86	27.2
**DHL3**	I feel confident making health-related decisions based on information from digital health services.	316	3.56	1.10	0	0.0	62	19.6	105	33.2	60	19.0	89	28.2
	**Access Barriers**													
**DHB1 (R)**	*Physical limitations such as vision or dexterity problems make it difficult for me to use digital health applications.*	316	2.80	1.04	57	18.0	35	11.1	140	44.3	82	25.9	2	0.6
**DHB2 (R)**	*I worry that digital health technologies may not work*	316	2.93	1.15	35	11.1	83	26.3	100	31.6	65	20.6	33	10.4

	reliably when I need them.												
**DHB3 (R)**	*My limited experience with technology makes it difficult for me to use digital health services independently.*	316	2.35	1.11	103	32.6	52	16.5	110	34.8	49	15.5	2	0.6
**Institutional Trust**														
**DHT1**	I trust the information provided by official digital health services offered by the Ministry of Health.	316	3.16	0.89	14	4.4	34	10.8	181	57.3	61	19.3	26	8.2
**DHT2 (R)**	*I am concerned about the privacy and security of my personal health information when using digital health applications.*	313	2.53	0.77	35	11.2	96	30.7	165	52.7	16	5.1	1	0.3

Note. N = number of valid responses. Mean and SD reported after reverse coding. (R) = item reverse-coded (1→5, 2→4, 3→3, 4→2, 5→1) prior to analysis. Highlighted rows indicate reverse-coded items.
